# Multispectral diffuse reflectance can discriminate blood vessels and bleeding during neurosurgery based on low-frequency hemodynamics

**DOI:** 10.1117/1.JBO.25.11.116003

**Published:** 2020-11-11

**Authors:** Audrey Laurence, Alain Bouthillier, Manon Robert, Dang K. Nguyen, Frédéric Leblond

**Affiliations:** a Polytechnique Montréal, Department of Engineering Physics, Montréal, Québec, Canada; b Centre de Recherche du Centre Hospitalier de l’Université de Montréal, Montréal, Québec, Canada; c Centre Hospitalier de l’Université de Montréal, Division of Neurosurgery, Montréal, Québec, Canada; d Centre Hospitalier de l’Université de Montréal, Division of Neurology, Montréal, Québec, Canada

**Keywords:** neurosurgery, tissue optics, spectroscopy, diffuse reflectance, hemodynamics

## Abstract

**Significance:** The practicality of optical methods detecting tissue optical contrast (absorption, elastic and inelastic scattering, fluorescence) for surgical guidance is limited by interferences from blood pooling and the resulting partial or complete inability to interrogate cortex and blood vessels.

**Aim:** A multispectral diffuse reflectance technique was developed for intraoperative brain imaging of hemodynamic activity to automatically discriminate blood vessels, cortex, and bleeding at the brain surface.

**Approach:** A manual segmentation of blood pooling, cortex, and vessels allowed the identification of a frequency range in hemoglobin concentration variations associated with high optical signal in blood vessels and cortex but not in bleeding. Reflectance spectra were then used to automatically segment areas with and without hemodynamic activity as well as to discriminate blood from cortical areas.

**Results:** The frequency range associated with low-frequency hemodynamics and respiratory rate (0.03 to 0.3 Hz) exhibits the largest differences in signal amplitudes for bleeding, blood vessels, and cortex. A segmentation technique based on simulated reflectance spectra initially allowed discrimination of blood (bleeding and vessels) from cortical tissue. Then, a threshold applied to the low-frequency components from deoxyhemoglobin allowed the segmentation of bleeding from vessels. A study on the minimum acquisition time needed to discriminate all three components determined that ∼25  s was necessary to detect changes in the low-frequency range. Other frequency ranges such as heartbeat (1 to 1.7 Hz) can be used to reduce the acquisition time to few seconds but would necessitate optimizing instrumentation to ensure larger signal-to-noise ratios are achieved.

**Conclusions:** A method based on multispectral reflectance signals and low-frequency hemoglobin concentration changes can be used to distinguish bleeding, blood vessels, and cortex. This could be integrated into fiber optic probes to enhance signal specificity by providing users an indication of whether measurements are corrupted by blood pooling, an important confounding factor in biomedical optics applied to surgery.

## 1 Introduction

The practicality and application scope of optical methods in surgery are often limited by interferences from blood pooling. This includes fiber optics and camera-based macroscopic imaging applications using biomedical optics instrumentation to guide interventions based on the optical tissue contrast from diffuse reflectance (absorption, elastic scattering), fluorescence, and vibrational spectroscopy (inelastic scattering). This is because bleeding can lead to blood pooling within the imaging field, which in turn represents an important confounding factor since hemoglobin is often the most important chromophore in tissues. Clinical implementation problems then arise from the practical limitations associated with the impossibility to clear pooled blood during fast-paced and oftentimes complex surgical procedures. Another set of issues can arise from the need to discriminate blood vessels from other tissue types and provide this information to surgeons to limit hemorrhage.

Fiber optic probes are used in a variety of biomedical applications ranging from pathology identification to surgical guidance. In the case of brain biopsy procedures, probes have been developed to locate areas with higher significance for pathology diagnosis, by avoiding collecting tissue in necrotic areas^1^ or to ensure the safety of the procedure, by avoiding the rupture of blood vessels located near the biopsy needle.^2^ Preventing hemorrhage during brain biopsy procedures is critical since the risk of morbidity and mortality caused by intracranial hemorrhage can go up to 16% and 3%, respectively.^3^ Optical technologies allowing blood vessel detection in neurosurgical applications using intrinsic contrast include optical coherence tomography,^2^ diffuse reflectance spectroscopy detecting hemoglobin absorption contrast,^1^^,^^4^^,^^5^ and its pulsatile motion,^6^ near-infrared spectroscopy,^7^ and laser Doppler imaging.^8^^–^^11^

This paper presents a technique using multispectral reflectance signals to identify blood vessels located at the brain surface and differentiate them from bleeding that could have occurred in the neurosurgical cavity. The technique segments blood vessels, bleeding, and brain cortex in multispectral images acquired during epilepsy surgery. The segmentation algorithm is based on the absorption spectrum of hemoglobin to detect blood and exploits the fact that temporal variations in oxygenated hemoglobin concentration associated with blood flow are present only in blood vessels and not in bleeding. This temporal frequency detection increases the specificity of blood vessel detection compared to reflectance techniques based only on absorption spectra. A proof-of-principle is presented in this paper using a multispectral imaging system to detect hemodynamic activity during neurosurgery in three patients.

## 2 Methods

### 2.1 Multispectral Imaging System

A custom multispectral imaging system connected to a neurosurgical microscope (OPMI Pentero, Zeiss) was used to image the exposed cortex of a patient during epilepsy surgery.^12^ The system integrates a snapshot multispectral camera (IMEC, Leuven, Belgium) composed of 4×4 arrays of band-pass filters disposed over a charged-coupled device (CCD) chip of 1024×2048  pixels, leading to raw images of 16 spectral bands (480 to 630 nm with a 15-nm spectral resolution) of 256×512  pixels. The camera was operated at 20 frames per second (fps) to allow sufficient signal-to-noise ratio (SNR) and recordings were performed for 8 min for a total of 9600 images. The white light source (Superlux, 300 W xenon lamp) integrated into the microscope was used to continuously illuminate the brain surface during the imaging session. The microscope working distance was ∼25 cm and the total microscope magnification was set at 1.5×. The lenses in the camera adaptor led to a resulting magnification of 4.7, an imaging field of view of $∼13\;{\mathrm{cm}}^{2}$, and a spatial resolution ∼0.1  mm. Electrocorticography (ECoG) electrodes were placed on the patient’s brain but electrophysiological data were not used as part of the work presented here. Informed consent was obtained from the patient and the Centre Hospitalier de l’Université de Montréal (CHUM) ethics review board approved the research protocol.

The dataset is composed of acquisitions performed during epilepsy surgery in three patients where regions of accumulated blood were noticeable in the field of view. The dataset of patient 1 exhibited a clear drop of blood and was chosen to design the segmentation technique described in Secs. 2.2 and 2.3. Patients 2 and 3 exhibited areas of accumulated blood and were selected to demonstrate the technique’s capabilities to segment blood, blood vessels, and cortex in different patients. The total number of acquisitions performed with the multispectral imaging system reached 15 patients, but only three presented that spontaneous accumulation of blood and were included in the study.

Calibration and biophysical modeling procedures were applied to the spatial–temporal–spectral patient datasets resulting in a time-course of oxygenated hemoglobin (HbO) and deoxygenated hemoglobin (HbR) concentration variations at the surface of the cortex. A detailed description of the algorithms and model parameters can be found in Ref. 12. Briefly, spectral and spatial calibrations were initially performed to account for the system response by normalization using a dataset measured on a reflectance standard (Spectralon, Labsphere). Pixels attaining the detector maximum intensity were associated with specular reflections and were excluded from the analysis. Then, a spatial registration algorithm was implemented using the MATLAB^®^ Medical Image Registration Toolbox.^13^ That procedure was applied to each image, resulting in a dataset where the mechanical motion of the brain due to breathing and heartbeat was removed. A modified Beer–Lambert law was applied to all images on a pixel-by-pixel basis to compute the relative concentration changes of HbO and HbR.^14^ The differential pathlength factor used in the modified Beer–Lambert law was computed using estimates from the literature for the absorption and scattering coefficients $\left(\mu_{a},\mu_{s}^{\prime }\right)$ in brain.^15^ Parameters and equations used to model absorption and scattering coefficients are detailed in Sec. 2.3.1.

### 2.2 Hemodynamic Activity Characteristics of Cortex, Blood Vessels, and Bleeding

Following the application of the preprocessing steps, the dataset consisted of videos (9600 images) of brain hemodynamic activity, i.e., time-sequences showing the concentration variations of HbO and HbR for each of the 256×512  pixels. A Fourier transform was applied to the concentration variation of HbO within each pixel to obtain the frequency profile of the hemodynamic activity from 0 to 10 Hz with a resolution of 0.0024 Hz. To avoid confusion, in this paper, the term “temporal frequencies” refers to the frequency spectra resulting from the Fourier transform (Hz). Otherwise, the term “spectra” refers to the optical spectrum of the diffuse reflectance (nm). A standard normal variate (SNV) normalization was applied to temporal frequencies so that the relative distribution of components was independent of absolute power density.

The patient 1 dataset showed a blood drop within the imaging field of view that slowly accumulated in the top-left corner of the image and started dripping toward the bottom of the image. The time frames clearly exhibiting the blood drop (#4800 to 8000) were selected to evaluate the temporal and spectral characteristics of the three different components of the exposed brain: blood vessels, cortex, and bleeding. For patients 2 and 3, no apparent movement of blood was noticed and 4000 frames were selected for data processing. Manual segmentation of the three physiological components was performed within those time frames to identify some regions for which the nature of the imaged tissue could be ascertained. Bleeding was identified from areas of dense accumulated blood; blood vessels with clear boundaries including arteries and veins were selected; clear areas of cortex tissue were circled. The temporal frequency profiles of all pixels within each segmented area were averaged to visualize the temporal frequency profile of each component.

The temporal frequency interval of 0.03 to 0.3 Hz was identified as exhibiting large differences between blood vessels, bleeding, and cortex mainly due to the respiration rate that has a noticeable effect in HbO concentration trends and was set by the medical team to 0.23, 0.2, and 0.22 Hz for the three patients, respectively. Temporal frequency spectra were averaged over the 0.03 to 0.3 Hz range for each pixel of the segmented components and a Kruskal–Wallis one-way analysis of variance between regions was performed for the frequency range. A threshold value corresponding to the 75th percentile of the bleeding component was selected for each patient to determine the lower limit value of hemodynamic activity intensity, used in a semiautomatic segmentation algorithm.

### 2.3 Segmentation Algorithm Using Reflectance Spectra and Hemodynamic Activity

Using information obtained from Sec. 2.2, a semiautomatic segmentation technique was developed to identify blood vessels, bleeding, and cortex in the image. Compared to existing methods detecting the presence of blood vessels based on the presence of hemoglobin absorption signature in acquired reflectance spectra, this new method combines the spectral detection of hemoglobin with the temporal frequency characteristics of HbO time-response. This allows more specific discrimination between dynamic blood (blood vessels supplied in oxygen) and static blood that has accumulated.

The segmentation technique is performed in three steps, detailed in the following sections. For step one, pixels with a reflectance spectrum indicating the presence of hemoglobin are identified as blood, including here bleeding and blood vessels. For step two, pixels with HbO temporal frequency profiles indicating strong low-frequency components are identified as dynamic (cortex, blood vessels). Other pixels are identified as static (bleeding, skull, and other structures). Step 3 consists of combining the information of steps 1 and 2 to categorize pixels into bleeding, blood vessels, or cortex. Each of the three segmentation steps is detailed in the following sections of the paper.

#### 2.3.1 Segmentation of blood based on diffuse reflectance spectra

Calibrated diffuse reflectance spectra in images were compared to simulated reflectance spectra of blood and cortex to determine the nature of each pixel. Reflectance spectra of blood and cortex were simulated using estimations of the absorption and scattering coefficients. The following model was used for the absorption coefficient ($\mu_{a}$)^15^

$$\mu_{a}=BS\mu_{a,\mathrm{HbO}}+B(1-S)\mu_{a,\mathrm{HbR}}+\mu_{a,0}\;({\mathrm{mm}}^{-1}),$$ (1)

where $\mu_{a,\mathrm{HbO}}$ and $\mu_{a,\mathrm{HbR}}$ are the theoretical absorption coefficient values of HbO and HbR^16^ in ${\mathrm{mm}}^{-1}$, S is the oxygen saturation of blood (no unit), B is the proportion of vessels in the cortex (no unit), and $\mu_{a,0}$ is a baseline absorption coefficient (${\mathrm{mm}}^{-1}$). The parameters B and S were first determined from the literature^15^ and then optimized for each patient based on the resulting modeled reflectance spectra. A high saturation (0.99 and 0.98) was selected to model blood to capture both bleeding and blood vessels with this modeling, and saturation of 0.90 was selected for cortex to take into account both veins and arteries capillaries in the cortex tissue. Parameters used for each patient are summarized in Table 1. For the reduced scattering coefficient ($\mu_{s}^{\prime }$), the equation $\mu_{s}^{\prime }=2.2{\left(\lambda /500\right)}^{-0.66}\;({\mathrm{mm}}^{-1})$ was used for blood and $\mu_{s}^{\prime }=2.42{\left(\lambda /500\right)}^{-1.661}\;({\mathrm{mm}}^{-1})$ for cortex^15^ for all patients, where λ is the wavelength in nanometers.

**Table 1:** Saturation, blood content, and baseline absorption coefficient parameters to model absorption coefficient of blood and cortex.

	Blood		Cortex		
	B	S	B	S	$\mu_{a,0}$ (${\mathrm{mm}}^{-1}$)
Patient 1	1	0.99	0.5	0.9	0.001
Patient 2	1	0.99	0.5	0.9	0.01
Patient 3	1	0.98	0.5	0.9	0.01

The modeled diffuse reflectances for blood and for cortex were estimated using the standard diffusion approximation for the wavelength range of interest (480 to 630 nm).^14^ These modeled reflectance spectra $R_{\text{blood}}$ and $R_{\text{cortex}}$ were fitted to the experimental reflectance spectra $R_{d}$ using a least-squares method to obtain the proportions of blood and cortex representing each pixel. These coefficients $a_{\text{blood}}$ and $a_{\text{cortex}}$ were obtained by minimizing the equation

$$\sum {\left\{R_{d}(\lambda )-[a_{\text{blood}}R_{\text{blood}}(\lambda )+a_{\text{cortex}}R_{\text{cortex}}(\lambda )]\right\}}^{2},$$ (2)

for each pixel. The coefficients $a_{\text{blood}}$ and $a_{\text{cortex}}$ were then expressed between 0 and 1. Based on the obtained data, pixels with a value of $a_{\text{blood}}>0.8$ were considered as blood, and pixels with $a_{\text{cortex}}>0.75$ were considered as cortex to produce a binary segmented image. These thresholds were determined by hand to optimize the segmentation of the two components and were validated visually, by ensuring the blood component could capture as many small vessels as possible. The same threshold values were used for all three patients.

#### 2.3.2 Segmentation of blood vessels and cortex based on the hemodynamic activity profile

The whisker plots from manually segmented regions described in Sec. 2.1 highlighted the temporal frequency range of 0.03 to 0.3 Hz with significant differences between bleeding, cortex, and blood vessels. The values of the SNV-normalized frequency amplitude were averaged in the 0.03 to 0.3 Hz range for each pixel to obtain a metric representing the hemodynamic activity level across the image. A threshold corresponding to the 75th percentile of the blood component was applied to this metric to separate pixels in two categories: with hemodynamic activity (dynamic) and without hemodynamic activity (static). The threshold values used were 1.84, 1.27, and 2.31 for patients 1, 2, and 3, respectively.

#### 2.3.3 Combination of presegmented images for segmentation of blood vessels, bleeding, and cortex

Steps one and two of the segmentation algorithm led to binary images indicating blood/cortex and dynamic/static. Logical intersections between the binary images produced in Secs. 2.3.1 and 2.3.2 allowed segmenting the images in terms of bleeding, blood vessels, and cortex: (1) pixels being both marked as blood and with the presence of hemodynamic activity were identified as blood vessels, (2) pixels being marked as blood and without hemodynamic activity were identified as bleeding, and (3) pixels identified as cortex and with hemodynamic activity were identified as cortex.

## 3 Results

### 3.1 Hemodynamic Activity of Cortex, Blood Vessels, and Bleeding

**Fig. 1:**
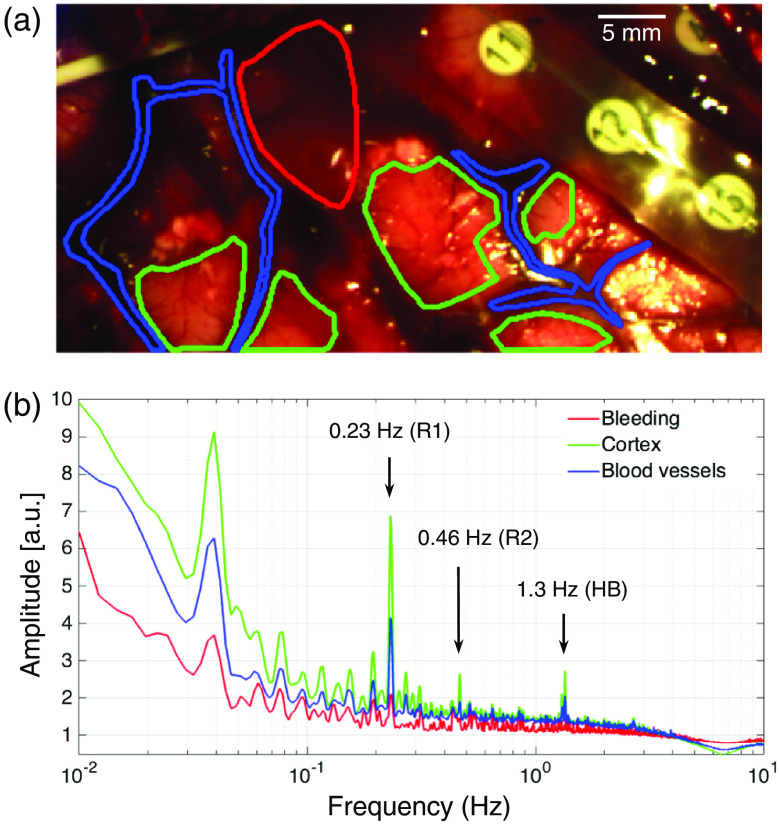
Hemodynamic activity profiles of cortex, blood vessels, and bleeding: (a) manual segmentation of three visually distinct regions: cortex (green), blood vessels (blue), and bleeding (red). ECoG electrodes, not used in this study, can be observed in the top right corner of the image. (b) Average SNV temporal frequency spectrum of HbO concentration for each of the three regions. Identified peaks correspond to respiration rate (R1), its second harmonic (R2), and heartbeat (HB).

Figure 1 shows the manual segmentation [Fig. 1(a)] and average temporal frequency spectrum [Fig. 1(b)] for patient 1. Averaged HbO concentration variations for each region of the brain exhibited an inverse trend (1/f) with respect to the temporal frequency f, which has been observed to be associated with spontaneous hemodynamic activity,^17^ combined with sharper and distinctive peaks at specific frequencies [Fig 1(b)]. The peak at ∼0.23  Hz corresponds to the respiratory rate for this patient, which was set by the medical team at 14 breathing cycles per minute during the surgical procedure. This peak was present in the cortex and vessel regions, but it had noticeably smaller amplitude within the blood drop. The second harmonic of the respiratory rate is also visible in the temporal frequency spectrum at ∼0.46  Hz, as well as the heartbeat at ∼1.3  Hz (79  beats/min), which are consistent with the values recorded by the physiological monitoring device during the procedure.

**Fig. 2:**
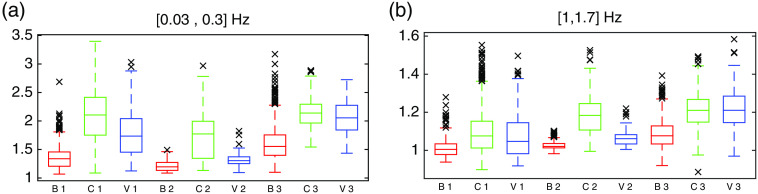
Distribution of the intensity of the SNV-normalized temporal frequency HbO spectra of each pixel comprised within the segmented components of bleeding (B), cortex (C), and blood vessels (V) for each patient (labeled 1, 2, and 3): (a) 0.03 to 0.3 Hz and (b) 1.0 to 1.7 Hz.

Whisker plots were produced to display the distribution of frequency intensity within all three segmented regions and for two frequency ranges exhibiting the most differences between regions: 0.03 to 0.3 Hz [Fig. 2(a)] associated with respiration rate and 1 to 1.7 Hz [Fig. 2(b)] associated with the heartbeat. Outside of these ranges (f<0.03 and f>1.7  Hz), data showed little differences in amplitude and less SNR. Amplitudes were noticeably lower for bleeding in all frequency ranges, except for the lowest frequencies (0 to 0.03 Hz) where the differences between regions were less apparent. A Kruskal–Wallis one-way analysis of variance between regions showed a p-value <0.01 for all frequency ranges. The temporal frequency amplitudes of pixels for cortex and blood vessels also appeared to be differentiable, with the largest observed differences in the 0.03 to 0.3 Hz range. The outliers in the whisker plots corresponded to pixels detecting high reflectivity (specular reflection) and were mostly associated with CCD saturation. Those pixels represented <0.7% of all data points in the 0.03 to 0.3 Hz range and <4% in the 1.0 to 1.7 Hz range. The performance of the brain motion correction algorithm was diminished for those high intensity pixels, which can explain the larger number of outliers in the frequency range containing the signal form heartbeat. For each patient, the bleeding region was the one with the lowest signal amplitude, and the cortex region appeared to have the highest signal amplitude. Average signal intensity varies from one patient to another, with patient 2 having the lowest. A threshold specific to each patient was then chosen to separate the bleeding component from cortex and blood vessels based on these intensity differences.

### 3.2 Segmentation of Blood Vessels, Bleeding, and Cortex

**Fig. 3:**
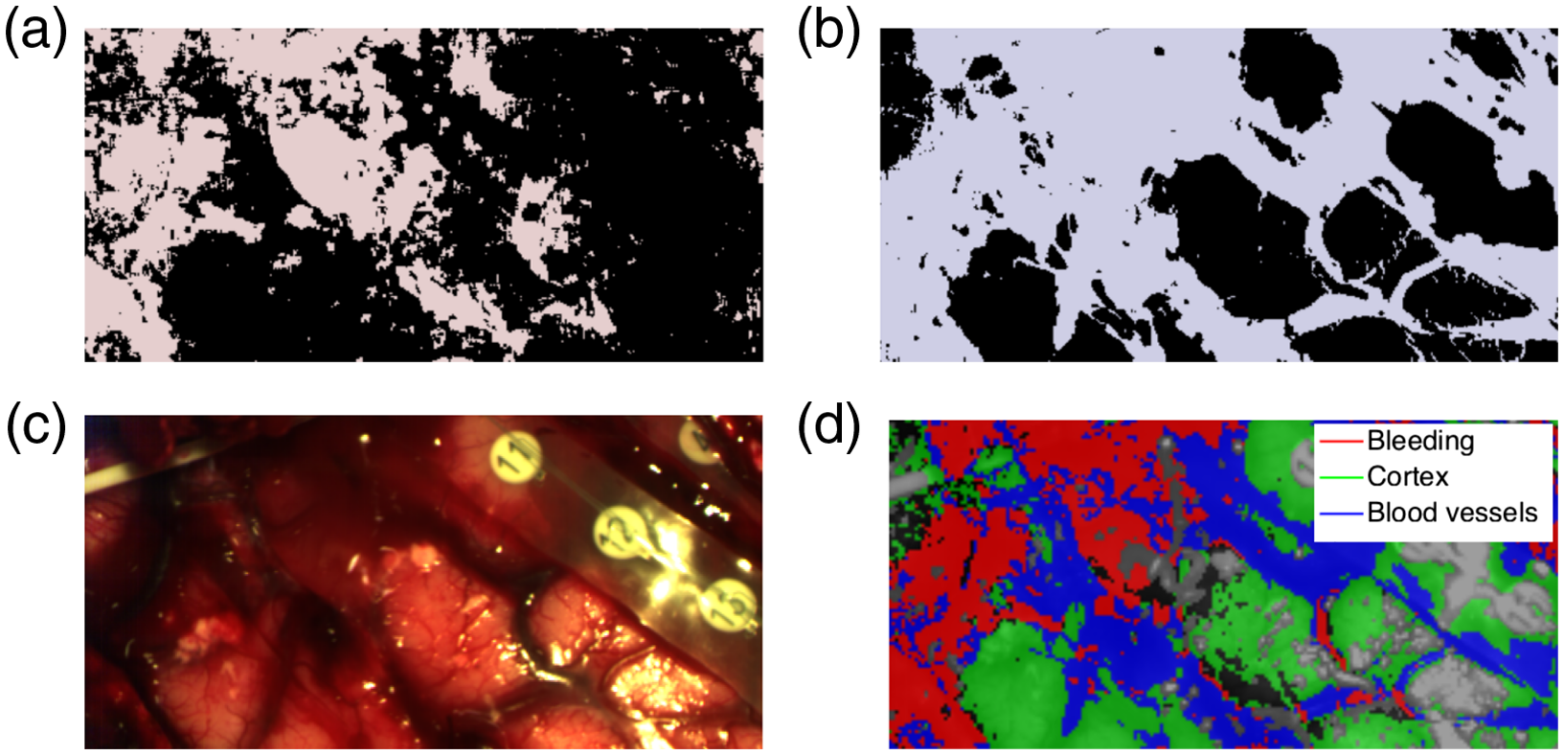
Semiautomatic segmentation of bleeding, blood vessels, and brain cortex in patient 1: (a) segmentation of regions with hemodynamic activity (black) and regions without hemodynamic activity (light red) using a method based on the temporal frequency of reflectance signals; (b) segmentation of cortex (black) and blood (light blue) using a method based on the reflectance spectrum; (c) color visualization of the region of interest; and (d) bleeding, blood vessels, and cortex segmentation using logical intersections of images in (a) and (b). Gray pixels indicate specular reflections.

Figure 3(a) shows the resulting segmented regions with hemodynamic activity (in black) and without activity (in light red) for patient 1. The blood drop at the image top in Fig. 3(c) and the blood accumulation toward the image bottom are identified as inactive by the algorithm and thus correspond to bleeding. Figure 3(b) displays the blood (light blue) and cortex (black) segmentation using the reflectance spectra-based segmentation algorithm. Both bleeding and blood vessels are identified as blood components by the algorithm. Figure 3(d) shows the final segmentation step, combining the results of Figs. 3(a) and 3(b). Bleeding is presented in red, blood vessels are in blue, and cortex in green. Gray pixels indicate saturated pixels due to specular reflections and black pixels were not classified in any category.

**Fig. 4:**
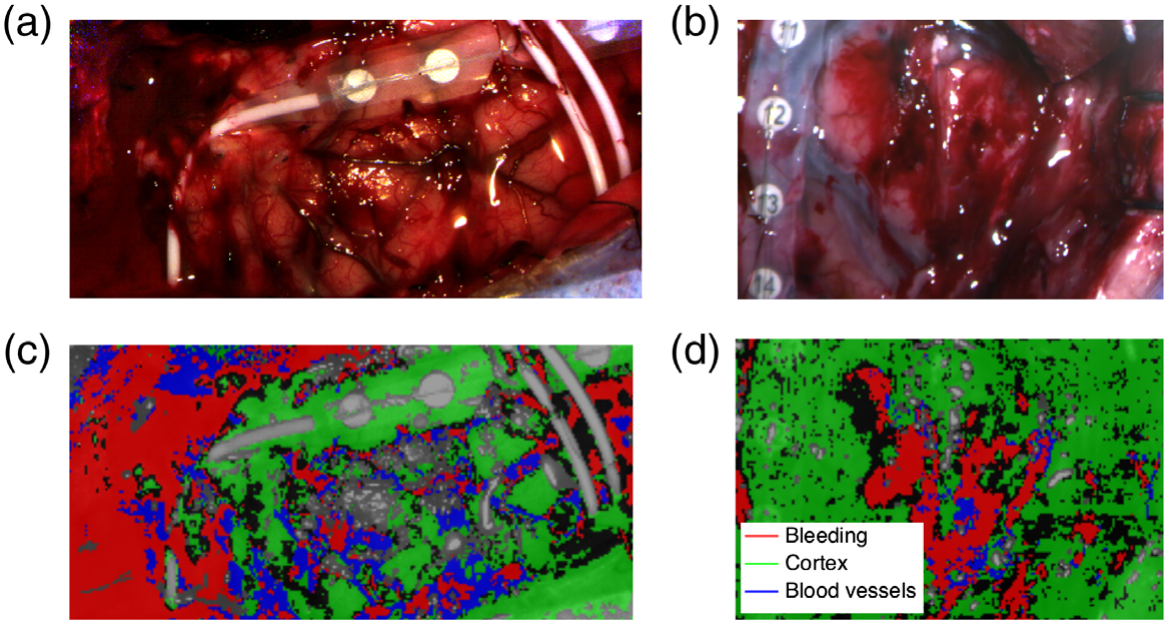
Bleeding, blood vessels, and cortex segmentation on two patients: (a), (b) color visualization of the region of interest for patient 2 and patient 3; (c), (d) bleeding, blood vessels, and cortex segmentation. Gray pixels indicate specular reflections.

Color visualization of the region of interest is presented for patients 2 [Fig. 4(a)] and 3 [Fig 4(b)], with the final segmentation results of blood vessels, cortex, and bleeding [Figs. 4(c)–4(d)]. Areas of blood pooling are identified as bleeding by the algorithm. Blood vessels are identified for patient 2, but only one deep blood vessel is present for patient 3’s region of interest and was not detected as a blood vessel by the algorithm. The areas corresponding to the plastic of the electrodes are associated with the largest errors in terms of performance for the registration algorithm used in the preprocessing phase. This implies that residues of the movement of the brain due to heartbeat and respiration rate appear in HbO time sequences and thus the areas are classified as “dynamic” by the algorithm.

### 3.3 Minimal Acquisition Time Analysis

The analysis presented in Fig. 3 for patient 1 was performed using 3200 frames acquired at 20 fps, corresponding to a total acquisition time of 160 s. However, such large acquisition times are not practical for surgical applications, which typically require much faster identification of vessels or blood pooling in a matter of seconds if not milliseconds. Considering that the lowest frequency within the interval displaying the most discriminatory potential (0.03 Hz) has a period of 33.3 s, the technique theoretically requires an acquisition time of around 30 s. An evaluation of the minimal acquisition time necessary to segment the image using the 0.03 to 0.3 Hz frequency range was performed by extracting sections of the dataset with a different number of frames and performing the segmentation algorithm on every section. Sections were formed by selecting n consecutive frames, with n ranging from 100 to 3600 by steps of 100 frames. The results were compared to reference values obtained with the full acquisition, i.e., with 3200 frames. The average temporal frequency spectrum intensities within the 0.03 to 0.3 Hz range were similar (<10% variation) to the reference values for acquisition times associated with >500  frames (25 s), and variations increased to >20% for an acquisition of 200 frames (10 s). The two-step automatic segmentation algorithm for these limited time-sequences further confirmed significant degradation of segmentation results at 500 frames.

## 4 Discussion

We presented a diffuse reflectance technique to detect blood vessels in brain surgery applications and discriminate them from bleeding that could occur during procedures such as biopsies and resection. The processing steps and instrumentation detailed in this paper were necessary in the context of multispectral imaging but could be simplified for fiber optic probe applications. This method could be performed with low-cost instrumentation (diodes and photodetectors) combined with standard reflectance data processing to detect hemoglobin concentration variations. The new semiautomatic segmentation technique visually illustrates the method capabilities to segment biological components, but the major improvement over current techniques using diffuse reflectance resides in its capabilities to distinguish bleeding from blood vessels, which is permitted by the dynamic reflectance measurements of the multispectral imaging system.

The segmentation algorithm based on reflectance signals [Fig. 3(a)] involved modeling of cortex and blood optical properties with optimization of blood content, blood saturation, and absorption coefficient. While we have optimized these parameters, we have used the same scattering coefficients for each patient. This variability across patients will be studied in future work to provide insight into the sensibility of the segmentation algorithm to specific optical properties parameters. The reflectance-based segmentation also involved selecting thresholds to segment blood from cortex areas, which was performed by optimizing segmentation in all three patients together. On the other hand, the segmentation algorithm using hemodynamic activity [Fig. 3(b)] was based on specific thresholds optimized for every patient individually, due to differences in intensity levels of the hemodynamic activity. Future work will include an extra calibration step to allow a more robust comparison between patient datasets ensuring intensity of the temporal frequency profiles is reproducible between acquisitions.

Although the reflectance modeling with the diffusion approximation is known to be inaccurate in high absorption tissue, we consider that for the presented method involving thresholds on the obtained proportions $a_{\text{blood}}$ and $a_{\text{cortex}}$, the uncertainties of modeling are not crucial to the technique. Moreover, the results show that the segmentation performs adequately even with the diffusion approximation, using the same thresholds for every patient. A detailed assessment of the errors of the standard diffusion approximation compared to the radiative transfer equation solutions could be performed but was deemed outside of the scope of the manuscript.

The segmentation performed using the 0.03 to 0.3 Hz frequency range exhibited the clearest separation between regions, but Fig. 2(b) shows that other frequency intervals could be used in the analysis to reduce the acquisition time. The range corresponding to the heartbeat (∼1.5  Hz) would allow the reduction of the acquisition time to few seconds, with the condition that the detection system allows sufficient SNR in this regime. Implementation of the technique in surgical applications such as brain biopsy needles^1^ or multimodal spectroscopy probes for *in situ* cancer detection^18^ might then necessitate acquiring data with fiber optic and high quantum efficiency spectrometers that have greater light sensitivity and transmission than the multispectral system used in this study.

We propose that mostly two frequency intervals could be selected to distinguish blood vessels from bleeding and from tissue: low-frequencies including the respiratory rate (∼0.2  Hz) and heartbeat (∼1.5  Hz). Respiratory rate offers higher SNR and permits a low sampling rate but necessitates ∼25  s acquisition time to successfully distinguish tissue types. Heartbeat frequencies would allow acquisition times of few seconds, but instrumentation would need to be optimized to ensure high SNR in this regime. For brain surgery procedures, the respiratory rate is set by the medical team and is stable over time, whereas the heartbeat varies from one patient to another but can be measured easily and used as an input parameter in the processing algorithms.

The presented method could also find applications in fiber optic probes for tissue identification such as cancer detection. Methods based on automatic classification algorithms for tissue diagnosis^18^ are sensitive to the presence of bleeding at the tip of the probe, which affects data quality. The knowledge of the presence of bleeding could help for example to build more robust classification models for tissue diagnostic in surgical interventions.
